# Supplementary material for: SumGNN: multi-typed drug interaction prediction via efficient knowledge graph summarization
Source: Bioinformatics. 2021 Mar 26;37(18):2988–95. doi: 10.1093/bioinformatics/btab207 (PMC10060701; doi:10.1093/bioinformatics/btab207)
Supplement: btab207_supplementary_data [file btab207_supplementary_data.pdf]

A Implementation Details

A.1 `SumGNN` Parameter Setup

We use the following hyperparameter set for `SumGNN` after random search on validation set. We use $1,024$-bits Morgan fingerprint for drug featurization. We set the subgraph to be 2-hops neighbors (i.e. $k = 2$). In the subgraph summarization module, we use weight matrix of size $d = 32$ for $\mathbf{W}_1$ and $\mathbf{W}_2$. The hidden dimension $\mathbf{h}_v^k$ is set to be $d = 32$. The relation matrix $\mathbf{r}$ is set to be 32. The edge pruning threshold is set to be $\gamma = 0$. The input hidden representation of each node is $d = 32$. The number of basis B in Eq. (3) is set to 8 as the performance do not change much when set from 4 to 16 and suffer from over-fitting with $B > 16$. We study the effect of key parameter d, γ and k in our experiment part (Section 4).

A.2 Training Details

Training Parameters. For both our method and baselines, the training parameters are set as follows unless specified.

We train the model for 50 epochs with batch size 256. Our model is optimized with ADAM optimizer (Kingma and Ba, 2014) of learning rate 5×10^{-3} with gradient clipping set to 10 under L2 norm. We set the L2 weight decay to 1×10^{-5}, the layer of GNN to 2 and set the dropout rate to 0.3 for each Layer in GNN.

Model Implementation and Computing Infrastructure. All methods are implemented in PyTorch[3] and the graph neural network modules are build on Deep Graph Library (DGL)[4]. The System we use is Ubuntu 18.04.3 LTS with Python 3.6, Pytorch 1.2 and DGL 0.4.3. Our code is run in a Intel(R) Core(TM) i7-5930K CPU @ 3.50GHz CPU and a GeForce GTX TITAN X GPU.

A.3 The Range for Tuning Hyper-parameters

We use grid search to determine hyper-parameters and list the search space of key hyper-parameters as follows.

Table 3: The range for tuning hyper-parameters. The bold numbers are the default settings.

Parameters	Range
Learning Rate	$[5 \times 10^{-4}, 1 \times 10^{-3}, \mathbf{5 \times 10^{-3}}, 1 \times 10^{-2}]$
Weight Decay	$[1 \times 10^{-6}, \mathbf{1 \times 10^{-5}}, 1 \times 10^{-4}, 1 \times 10^{-3}]$
Dropout	$[\mathbf{0.3}, 0.4, 0.5]$
Layers of GNN	$[1, \mathbf{2}, 3]$
d	$[8, 16, \mathbf{32}, 64]$
k	$[1, \mathbf{2}, 3, 4]$
B	$[4, \mathbf{8}, 12, 16, 24, 32]$

[3] `https://pytorch.org/`
[4] `https://www.dgl.ai/`

A.4 Baseline Setup

For the baselines, the settings are described as follows:

- **MLP**: We implement MLP with Pytorch with the Morgan fingerprint. We use a two-layer MLP and set the hidden dimension to 100 with dropout 0.3.
- **Node2vec**: We follow the officially released implementation from authors[5] and set the embedding dimension to 64.
- **Decagon**: We use DGL to implement the model. Following (Zitnik et al., 2018a), we set the number of GNN layers to 2 set the hidden dimension to 64 and 32 for two layers with a dropout rate of 0.1 and a minibatch size of 512.
- **GAT**: We use DGL to implement the model and set the hidden dimension to 64 with 4 attention heads, as we find that improving the number of heads will hurt the performance. We set the activation function to `LeakyReLU` with $\alpha = 0.2$.
- **Others**: We follow the officially released implementa- tion from the authors listed as follows:
  - **SkipGNN**: https://github.com/kexinhuang12345/SkipGNN.
  - **KG-DDI**: the neural model is based on code in https://github.com/rezacsedu/Drug-Drug-Interaction-Prediction, and the KG embeddings are trained via OpenKE toolbox https://github.com/thunlp/OpenKE.
  - **GraIL**: https://github.com/kkteru/grail.
  - **KGNN**: https://github.com/xzenglab/KGNN.

[5]https://github.com/aditya-grover/node2vec
